# C-reactive protein in predicting major postoperative complications are there differences in open and minimally invasive colorectal surgery? Substudy from a randomized clinical trial

**DOI:** 10.1007/s00464-017-5996-9

**Published:** 2017-12-27

**Authors:** Jennifer Straatman, Miguel A. Cuesta, Jurriaan B. Tuynman, Alexander A. F. A. Veenhof, Willem A. Bemelman, Donald L. van der Peet

**Affiliations:** 1 Department of Surgery, VU University Medical Center, De Boelelaan 1117 - ZH 7F020, 1081 HV Amsterdam, The Netherlands; 2 Department of Surgery, Antoni van Leeuwenhoek Hospital, Amsterdam, The Netherlands; 3 Department of Surgery, Academic Medical Center, Amsterdam, The Netherlands

**Keywords:** Minimally invasive surgery, Colorectal surgery, Postoperative complications, C-reactive protein, Quality control

## Abstract

**Background:**

In search of improvement of patient assessment in the postoperative phase, C-reactive protein (CRP) is increasingly being studied as an early marker for postoperative complications following major abdominal surgery. Several studies reported an attenuated immune response in minimally invasive surgery, which might affect interpretation of postoperative CRP levels. The aim of the present study was to compare the value of CRP as a predictor for major postoperative complications in patients undergoing open versus laparoscopic colorectal surgery.

**Methods:**

A subgroup analysis from a randomized clinical trial (LAFA-trial) was performed, including all patients with non-metastasized colorectal cancer. In the LAFA trial, patients were randomized to open or laparoscopic segmental colectomy. In a subgroup of 79 patients of the LAFA trial, postoperative assessment of CRP levels was conducted routinely preoperatively and 1, 2, 24 and 72 h after surgery.

**Results:**

Thirty-seven patients were randomized to the open group and 42 patients to the laparoscopic group. Major complications occurred in 19% of laparoscopic procedures and 13.5% of open procedures (*p* = 0.776). CRP levels rise following surgical procedures. In uncomplicated cases, the rise in CRP levels was significantly lower at 24 and 72 h following laparoscopic resection in comparison to open resection. No differences in CRP levels were observed when comparing open and laparoscopic resection in patients with major complications.

**Conclusion:**

In patients with an uncomplicated postoperative course, CRP levels were lower following minimally invasive resection, possibly due to decreased operative trauma. No differences in CRP were observed stratified for surgical technique in patients with major complications. These results suggest that CRP may be applied as a marker for major postoperative complications in both open and minimally invasive colorectal surgery. Future research should aim to assess the role of standardized postoperative CRP measurements.

Over the past decades, medical care for patients undergoing colorectal surgery has improved significantly with Fast-Track perioperative care and minimally invasive surgical techniques [1]. Minimally invasive techniques have proven to be superior to conventional open techniques in colorectal surgery for short-term outcomes, such as improved postoperative recovery and a reduced postoperative systemic immune response with possible concomitant inhibitory effect on tumour spread and metastasis. Long-term outcomes are similar in both groups [2–18].

Major complications after colorectal surgery, requiring invasive treatment, are reported in up to 19% of patients [1, 19, 20]. In search of improvement of patient assessment in the postoperative phase, C-reactive protein (CRP) is increasingly being studied as both an early marker for postoperative complications, as well as a safe discharge criterion [19, 21]. CRP levels rise following surgery and a peak is observed after 48 h. After 48 h, levels decrease in patients with an uncomplicated postoperative course. CRP levels were found to be significantly higher in patients with major complications compared to patients with no or minor complications. These differences are observed from as early as the second postoperative day, with a median time from surgery to diagnosis of complications of 7 days [19]. Multiple studies have found CRP levels on the third and fourth postoperative day to be predictive for major complications following major abdominal surgery [21, 22].

Several studies have assessed the use of CRP as an early marker for postoperative complications following colorectal surgery. Some studies included both open and minimally invasive procedures, but no statistical analysis was performed on the differences between surgical techniques in these studies [23–28]. Several other studies included only open procedures [29, 30] or only minimally invasive procedures [31], with similar results.

If the inflammatory and immune responses are attenuated in minimally invasive procedures in comparison to open procedures, this might affect interpretation of postoperative CRP levels and could imply a different cut-off should be applied for CRP as a marker for complications [32]. A recent meta-analysis showed no differences in CRP levels between open and minimally invasive surgery in patients that suffered postoperative complications. The study did suffer from selection bias, since gastric and oesophageal resections were only performed with open procedures, whereas gastric bypass surgery was only performed laparoscopically [22]. Additional evidence is necessary.

The aim of the present study was to compare postoperative CRP levels in patients undergoing open versus laparoscopic colorectal surgery within the context of a randomized trial in which CRP was routinely measured.

## Materials and methods

### Patients

The here presented study was performed as an observational study from the LAFA trial, “Perioperative strategy in colonic surgery; LAparoscopy and/or FAst track multimodal management versus standard care”. In the LAFA trial, patients were randomized to open or minimally invasive segmental colectomy and also randomized to standard or fast-track perioperative care. The full protocol was published previously [33]. Inclusion criteria consisted of (1) histologically confirmed malignancy or adenoma, (2) planned for elective segmental colectomy with curative intent, (3) age 40 to 80 years and (4) have an American Society of Anaesthesiologists (ASA) grade below IV. Exclusion criteria consisted of (1) a previous midline laparotomy, (2) emergency surgery, (3) planned ostomy or (4) immunosuppressive disease or medication. After obtaining informed consent, patients were randomized to open or minimally invasive elective segmental colectomy and fast-track or standard perioperative care. The design and primary results of the trial were previously published [1, 34].

Patients operated in the VU university medical center and Academic medical center in Amsterdam, the Netherlands, were included in this subgroup analysis. Patients in these hospitals received standardized postoperative blood sampling. In other participating centres, standardized measurements were not possible due to logistical reasons (i.e. samples would not reach laboratory in set time period).

Recorded data regarding baseline characteristics included age, gender, BMI, co-morbidities and American College of Anaesthesiologists (ASA) classification. Recorded clinical parameters included indication for operation, type of surgery, duration of surgery, clinical parameters, performed CT-scans, complications according to Clavien-Dindo classification and mortality.

Complications were graded according to a modified Clavien-Dindo classification, which grades complications according to the necessitated treatment [35, 36]. Minor complications, consisted of grade I and II, encompassing complications that require pharmacological treatment (i.e. antibiotics) and wound infections, treated by opening the wound at the bedside. Major complications consisted of grade III to V, encompassing all complications that require invasive treatment (i.e. percutaneous drainage or reoperation), ICU admission and including those leading to death. Mortality was defined as in-hospital mortality.

All patients received perioperative prophylactic intravenous antibiotics and thromboprophylaxis according to local protocol. Treatments of major complications were classified as reoperations, radiological interventions such as percutaneous drainage and intensive care admission.

### Design

Patients included in the LAFA-trial were randomized between laparoscopic or open segmental colectomy and fast-track or standard postoperative care in a two by two balanced factorial design, using an online randomization tool. The study was conducted in accordance with the principles of the declaration of Helsinki and the protocol approved by local medical ethics review boards (protocol number: NTR222). All patients provided written informed consent prior to inclusion in the study. The full protocol of the study was previously published [1, 34].

Patients that were treated in the VU University medical center and Academic Medical Center in Amsterdam underwent additional measurements of CRP levels. Samples were collected routinely preoperatively and 1, 2, 24 and 72 h after surgery. These measurements were planned alongside the original protocol and assessed and approved by the medical ethics committee. CRP data were compared for open and laparoscopic techniques. CRP samples were analysed by immunoturbidimetric methods, using the BM/Hitachi 705 (Boehringer, Mannheim, Germany).

All patients received similar postoperative assessment. Patients had daily assessment of clinical parameters (i.e. heart rate, blood pressure, temperature, pain, ileus). Upon clinical deterioration additional biochemical testing was performed (regardless of the standardized testing). Imaging was performed with computed tomography scans with oral and rectal contrast.

### Statistical analysis

Statistical analysis was conducted in SPSS version 19.0 (SPSS Inc. Chicago, IL, USA). Continuous variables with normal distributions were presented as means and standard deviations. Medians and interquartile ranges were used as a central tendency for continuous variables with abnormal distributions. Categorical data were expressed with percentage frequencies. Comparison between the open and minimally invasive group was conducted with Student’s *T* test or Mann–Whitney-U as appropriate for continuous variables. Chi-square tests were used for comparison of categorical data. A value of *p* < 0.05 was considered statistically significant. Logistic regression techniques were applied to determine confounders and effect-modifiers for major complications.

## Results

Eighty-one patients were assessed for eligibility. For this study, two patients were excluded since they declined extra blood sampling due to needle-phobia. Seventy-nine patients were randomized. Thirty-seven patients were randomized to an open procedure and 42 patients were randomized to a laparoscopic procedure in a 2 × 2 factorial design. The original trial also randomized patients to standard or fast-track perioperative care. In the laparoscopic group, 19 patients were randomized to fast-track care and 23 patients were randomized to standard care. In the open group, 17 patients received Fast Track care and 20 patients received standard care.

**Fig. 1:**
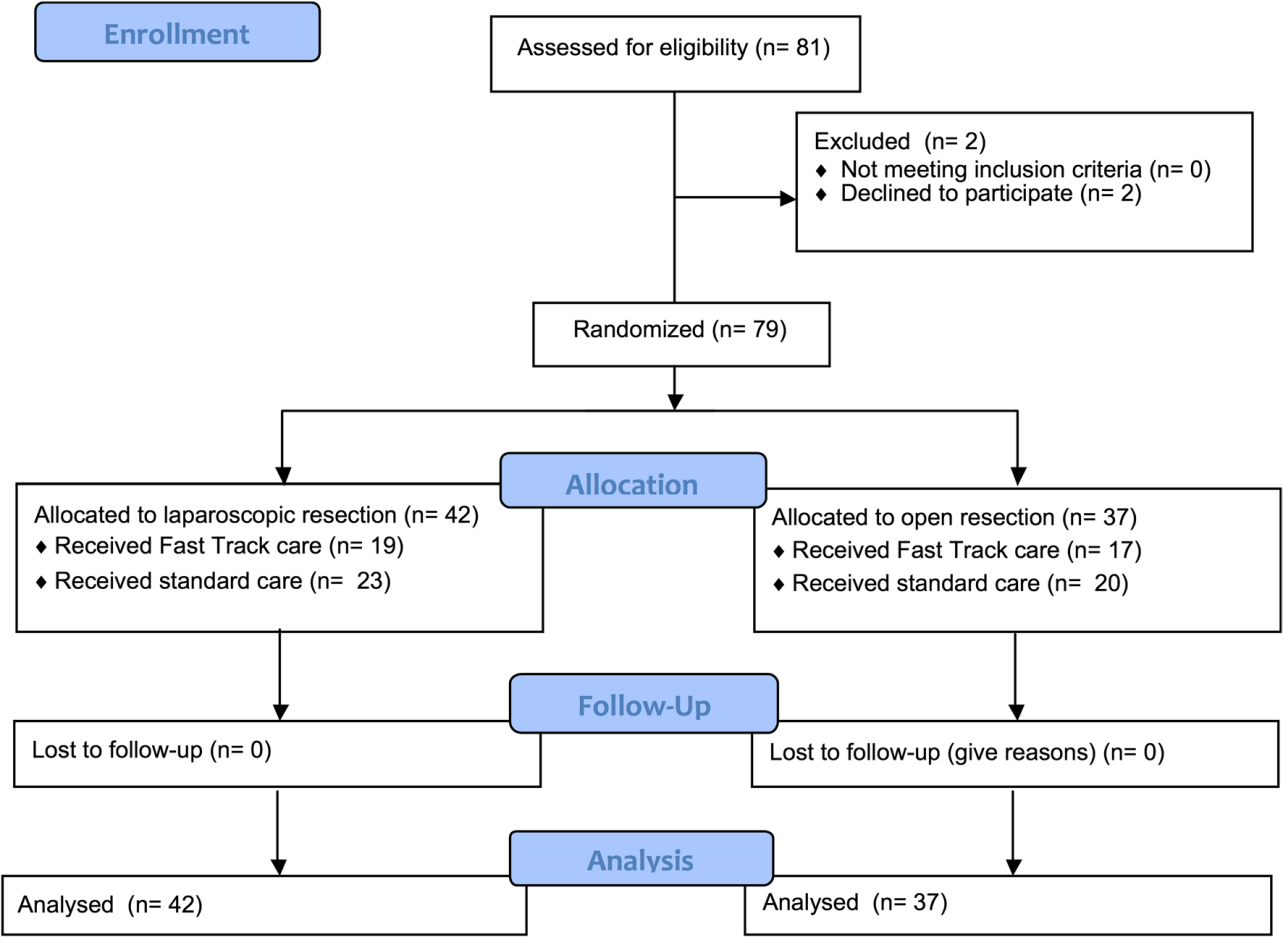
Flow chart for inclusion and analysis of patients

A flow chart for patient inclusion is depicted in Fig. 1. Baseline characteristics are depicted in Table 1. No statistically significant differences were observed between the open and laparoscopic group for patient characteristics. 94.7% of samples were collected and analysed on time, taking into account accrual times as described in the study protocol. Missing values were mainly caused by delay; samples were dismissed if they arrived at the laboratory outside the predetermined time interval. These samples were not analysed, and not included in the analysis for that measurement. (i.e. four patients excluded for analysis of 72-h samples).

**Table 1:** Baseline characteristics

Parameter	Open	Minimally invasive	*p* value
Patients (*n*)	37	42	
Gender male (%)	26 (69%)	23 (62.2%)	0.52
Age years (mean ± SD)	66.3 ± 12	66.7 ± 9.7	0.86
Body mass index (BMI) mean ± SD	25.8 ± 4.3	25.3 ± 3.3	0.59
ASA-classification			
I	9 (24.3%)	14 (33.3%)	0.50
II	24 (56.8%)	24 (57.2%)	
III	6 (16.2%)	4 (9.5%)	
IV	1 (2.7%)	0	
Comorbid disorders	27 (73%)	29 (69%)	0.70
Operative details			
Surgery type			
Right hemicolectomy	20 (54.1%)	15 (35.7%)	0.47
Transverse colectomy	1 (2.7%)	3 (7.1%)	
Left hemicolectomy	3^a^ (8.1%)	5 (11.9%)	
Sigmoid resection	11 (29.7%)	14 (33.3%)	
Rectum resection	2 (5.4%)	5 (11.9%)	
Duration of surgery min (mean ± SD)	130 (98–173)	191 (160–220)	< 0.001
Blood loss	230 (150–400)	80 (0–150)	< 0.001
Postoperative complications			
Uncomplicated^a^	28 (75.7%)	29 (69%)	0.47
Minor complication	4 (10.8%)	5 (11.9%)	0.78
Major complication	5 (13.5%)	8 (19.1%)	0.51
Hospital stay [median days (IQR)]			
Uncomplicated	6 (4–9)	4 (3–7)	0.01
Minor complication	10 (8–19)	14 (7–16)	0.99
Major complication	18 (11–72)	23 (13–40)	0.94
30-day mortality	1	–	0.29

Student’s *T* test was applied if the mean and standard deviation (SD) are depicted, being normal distributions. Mann–Whitney-*U* tests were applied if median and interquartile ranges (IQR) are depicted, being non-normal distributions

*ASA* American Society of Anaesthesiologists

^a^Chi-square test with post hoc Bonferroni analysis

### Surgical procedures

All patients underwent elective segmental colectomy for cancer. No significant differences were observed for the frequency of performed type of colectomy in the open or minimally invasive group (i.e. sigmoid resection, right or left hemicolectomy). Duration of surgery was significantly longer and blood loss was significantly less in the laparoscopy group (*p* < 0.001). In one patient, the procedure was converted to an open colectomy because of bulky tumour with ingrowth in the abdominal wall. Analysis was performed according to the intention to treat principle and this patient was analysed in the laparoscopy group.

### Postoperative complications

Complications were observed in 27.8% of patients. Major complications were observed in eight laparoscopy patients (19.1%) and in five patients who underwent an open procedure (13.5%). Minor complications were observed in 11.9% of laparoscopic procedures and 10.8% of open procedures. No differences in complication rates were observed for open and laparoscopic procedures (*p* = 0.540 for minor complications, *p* = 0.351 for major complications).

An overview of complications is depicted in Table 2. In patients with an uncomplicated postoperative course, following both open and minimally invasive procedures, hospital stay was significantly shorter in comparison to patients with a complicated postoperative course. Hospital stay was significantly longer in complicated patients and did not differ between open and laparoscopic procedures, as depicted in Table 1.

**Table 2:** Overview of all postoperative complications

Complication	Open	%	Minimally invasive	%	*p* value
Patients (*n*)	37		42		
Anastomotic leak	3	8.1	3	7.1	0.87
Surgery	2		1		
Percutaneous drainage	1		2		
Prolonged postoperative ileus	2	5.4	6	14.3	0.19
Surgery	–		3		
Wound/stoma problem	3	8.1	2	2.5	0.54
Surgery	–		–		
Non-abdominal					
Pneumonia	2	5.4	2	4.8	0.90
Cardiac complications	2	5.4	2	4.8	0.90

Cardiac complications occurred after the fifth postoperative day in both affected patients

### C-reactive protein

CRP data were collected routinely preoperatively (baseline) and 1, 2, 24 and 72 h postoperatively. No differences in CRP levels were observed between the different postoperative care groups, being standard versus fast track care, and minimally invasive versus open procedures, with a median CRP at 72 h of 124 in the standard care group and 96 in the fast-track group (*p* = 0.137). CRP levels measured after diagnosis of a complication were excluded (Table 3).

**Table 3:** Median CRP values and interquartile ranges in open and minimally invasive surgery

CRP	Open					
	Overall		Uncomplicated		Major complication	
	CRP (mg/L)	IQR	CRP (mg/L)	IQR	CRP (mg/L)	IQR
Preop	4	3–10	3	3–9	4	2–11
1 h	7	3–13	10	3–15	3	2–11
2 h	4	3–13	5	3–16	3	2–11
24 h	164	102–200	158	92–207	171	133–198
72 h	126	94–170	123	89–153	170	138–229

CRP	Minimally invasive					
	Overall		Uncomplicated		Major complication	
	CRP (mg/L)	IQR	CRP (mg/L)	IQR	CRP (mg/L)	IQR
Preop	3	3–17	3	2–19	3	2–17
1 h	3	3–15	3	2–19	3	2–11
2 h	3	3–17	4	3–22	3	2–14
24 h	99	53–166	96	46–152	137	3–190
72 h	84	43–152	78	32–138	125	56–430

CRP	Open versus minimally invasive colorectal resection	
	Uncomplicated	Major complications
	*p* value	*p* value
Preop	0.89	1.00
1 h	0.31	0.88
2 h	0.35	0.83
24 h	**0.01**	0.64
72 h	**0.05**	0.52

Significant differences are highlighted in bold

**Fig. 2:**
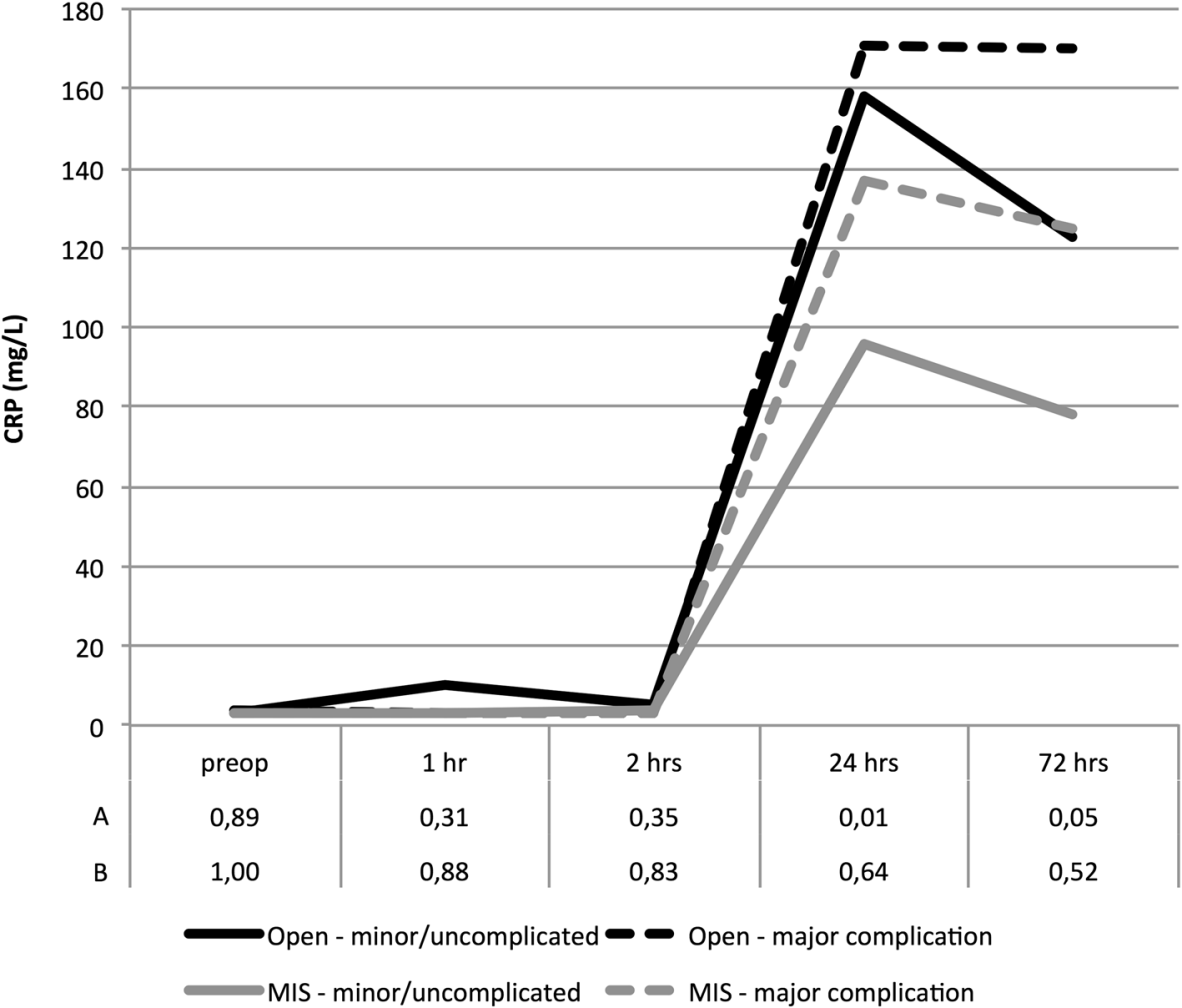
Postoperative median CRP levels in patients operated with minimally invasive or open techniques, stratified for major complications. Row A depicts *p* values for differences between open and minimally invasive surgery in patients with an uncomplicated or minor complicated postoperative course. Row B depicts *p* values in patients with major complications. *p* values were calculated using Mann–Whitney-*U* tests

Data were stratified for patients with major complications in comparison to patients with no or minor complications, as depicted in Fig. 2. In concordance with previous analyses, CRP levels were significantly lower in patients who underwent a laparoscopic procedure. At 24 h postoperatively median CRP levels were 164 mg/L in the open group versus 99 mg/L in the minimally invasive group (*p* = 0.008).

Analysis was further stratified for postoperative complications, comparing patients with major complications to patients with no or minor complications. In patients who had no or minor complications, CRP levels were lower in patients who underwent a minimally invasive procedure compared to open procedures at 24 and 72 h after surgery (*p* < 0.05). Receiver operator characteristic ROC curve analysis for CRP levels 72 h postoperatively as a marker for major complications revealed an area under the curve of 0.674 (95% confidence interval 0.506–0.842). By determining the Youden-index, the optimal cut-off was determined at CRP levels of 140 mg/L at 72 h postoperatively, with a negative predictive value of 90.2% and positive predictive value of 36.4%.

### Logistic regression analysis

Backward stepwise regression analysis was performed, to assess predictors for major complications. The following parameters were assessed in the model: laparoscopic versus open surgery, CRP levels 72 h postoperatively, sex, age and ASA-classification. The model is depicted in Table 4. In the primary multivariate regression model, with 5 variables in the equation, CRP levels at 72 h were not predictive for major complications. Following backward stepwise logistic regression, with a cut-off at *p* = 0.1, only CRP levels 72 h after surgery were found to be a significant predictor for major postoperative complications.

**Table 4:** Logistic regression analysis for major complications

Variables in the equation	B	S.E.	*p* value	Exp (B)
3a: Primary model				
Access: open versus laparoscopy	− 0.391	1.082	0.718	0.677
CRP 72 h	0.001	0.004	0.758	1.001
CRP 72 h by access	0.005	0.006	0.33	1.005
Female sex	− 0.212	0.796	0.79	0.809
ASA class I	Ref.		0.862	
ASA class II	− 0.597	0.909	0.511	0.55
ASA class III	− 1.288	1.541	0.403	0.276
ASA class IV	22.2	40,192	0.999	4,414,812
Age	0.032	0.004	0.758	1.032
Constant	− 3.384	3.345	0.312	0.034
3b: Final model				
CRP 72 h	0.004	0.002	0.05	1.004
Constant	− 2.173	0.48	0.001	0.114

In the primary model, all variables were inserted. Following backward stepwise regression analysis CRP remained as only significant predictor for major complications, with the overall model *p* < 0.05

CRP was added in the model per increase of 1 point in CRP levels. i.e. a 10 mg/mL point rise in CRP levels would be an odds radio of 1.04

## Discussion

Regardless of the applied surgical techniques, open or minimally invasive colorectal resection, postoperative CRP levels can be used to predict or rule out major postoperative complications.

In this subgroup analysis of a randomized clinical trial comparing open and minimally invasive surgery for colorectal cancer, CRP levels in patients with an uncomplicated postoperative course or minor complication were significantly lower in the minimally invasive group compared to the open group at 24 and 72 h postoperatively, whereas in patients with major complications no differences were observed for CRP levels between the two different surgical approaches. Correction with backward logistic regression techniques further emphasized that there was no effect-modification or confounding by surgical approach in the relationship between CRP levels and major postoperative complications. The determined cut-off for CRP as a marker for major postoperative complications was similar to a previously determined optimal cut-off based on a pooled analysis of 1427 patients, and the cut-off can be applied in both open and laparoscopic segmental colectomy [21].

The logistic regression model shows CRP levels at 72 h postoperatively to be predictive of postoperative complications. It should be noted the calculated AUC of 0.674 is lower compared to other similar studies [26], and may be explained by the smaller cohort size. In the primary multivariate regression model, CRP levels at 72 h were not predictive for major complications. This may be caused by having too many variables in the equation and low number of patients with major complications, giving a low sample size per variable. In a recent meta-analysis, including 1427 patients, CRP levels on the third postoperative day where found to be predictive for major complications with an AUC of 0.789, further supporting the use of CRP measurements as a tool for early detection of patients at risk of developing major complications [21].

CRP is a well-established marker for inflammation and has been studied as a predictor of postoperative complications; CRP was assessed separately as (1) a marker for anastomotic leak, (2) a marker for major complications and (3) a marker for all postoperative complications. Interestingly, multiple studies that assessed postoperative CRP levels included patients that were operated using both minimally invasive approach as well as conventional open approach. In these studies, no separate analysis was performed for differences in CRP levels between open and minimally invasive techniques [23–26, 37–39]. Only one study assessed both surgical techniques separately and found that average CRP levels were lower following minimally invasive surgery compared to open surgery, although the data were not stratified for adverse events [40]. Another study assessed CRP levels including only patients that underwent minimally invasive colorectal surgery and found a cut-off of 200 mg/L on postoperative day 3 as marker for major complications [31]. These results are similar to the study here presented. CRP levels at 72 h postoperatively were found to be predictive of major complications in patients that underwent open and minimally invasive surgery for colorectal cancer.

The results of the study here presented underpin the use of postoperative CRP levels as a marker for complications, regardless of surgical approach. The results further indicate that major complications may be developing earlier than we think. Higher CRP levels are seen in patients with major complications in comparison to patients with no or minor complications at 72 h postoperatively, whereas average time to diagnosis of complications is currently 5 days. A recent meta-analysis supports our findings; CRP levels were lower in patients that underwent minimally invasive surgery compared to open surgery if no complications occurred. As stated in the introduction this study suffered from selection bias [37]. Hence, we present this study based on randomized data.

Differences in CRP levels between open and minimally invasive surgery in patients with an uncomplicated or minor complicated postoperative course can be explained by the differences in the amount of operative trauma, leading to lower levels of acute phase proteins such as CRP and interleukin-6 [27, 41].

In patients with an uncomplicated postoperative course surgical trauma is the only stimulus for CRP synthesis. In patients with postoperative complications both surgical trauma and complications add up to increase CRP synthesis. Since no differences were observed in CRP levels between open or minimally invasive colorectal resection in patients with a postoperative complication, it is hypothesized that complications have a greater effect on CRP synthesis than surgical trauma, thereby diminishing the differences seen in uncomplicated patients.

This study was performed as a sub-study of the LAFA-trial, which investigated differences between both laparoscopic or open segmental colectomy and fast-track or standard care. The study has several limitations. Selection bias may not be omitted; since standardized measurements were performed only in two participating centers, these patients were included in this subgroup analysis. With standardized measurements only being available from two centers, the sample size is small.

The study was limited by its observational nature. CRP levels were measured routinely. In many observational studies, CRP levels are only determined on demand. A prospective clinical trial is underway from our department in order to further assess the role of standardized CRP measurements in a postoperative quality control algorithm for prediction of complications and as a safe discharge criterion [42].

In conclusion, CRP may be used as a marker for major postoperative complications in both open and minimally invasive colorectal surgery. Lower postoperative CRP levels are seen in patients who underwent minimally invasive surgery with an uncomplicated postoperative course and patients with minor postoperative complications, possibly due to a reduced amount of surgical trauma in minimally invasive surgery compared to open surgery. The inflammatory response in major complications is believed to exceed the effect of primary surgical trauma, and no differences in CRP levels are observed between open or minimally invasive surgery for colorectal cancer. Future research should aim to assess the role of standardized CRP measurements in patients undergoing major abdominal surgery.
